# Mycobacterium Avium Prosthetic Hip Infection on Abatacept Presenting as Fever of Unknown Origin

**DOI:** 10.7150/jbji.35703

**Published:** 2019-08-06

**Authors:** Rachel Sigler, Jessica R. Newman

**Affiliations:** 1 Department of Internal Medicine, University of Kansas Medical Center;; 2 Division of Infectious Diseases, University of Kansas Medical Center.

**Keywords:** MAC, joint infection, immunosuppression, prosthetic joint

## Abstract

Non-tuberculous mycobacteria (NTM) are well-described pulmonary pathogens in patients with underlying lung disease. Extra-pulmonary infections with NTM are rare. We describe a prosthetic hip infection with Mycobacterium avium complex. Increased immunosuppressing medications and number of total joint replacements are expected to increase the prevalence of NTM infections in the future.

## Introduction

Non-tuberculous mycobacteria (NTM) are abundant in the environment, present in soil and water sources worldwide. There are both rapidly-growing and slow-growing species. *Mycobacterium avium* complex (MAC) is the most common slow-growing NTM infection, with many asymptomatic carriers. NTM are commonly pathogenic in the HIV/AIDS population and in patients with chronic lung diseases including cystic fibrosis and bronchiectasis 4. Though septic arthritis and osteomyelitis is well described in *Mycobacterium tuberculosis*, joint disease with NTM is less common. Prosthetic joint infections (PJI) have rates following total hip arthroplasty (THA) of around 0.7%-2.4% 7 and are unusual to occur with NTM 1. They are even more rare with MAC. We present a case of an immunocompromised patient with MAC infection of a prosthetic hip joint. This case illustrates an infrequent complication of immunosuppressive therapy and the challenges of treating joint infection with NTM.

## Case Report

A 56-year-old male with a past medical history of seronegative rheumatoid arthritis (treated with abatacept 1000 mg every 4 weeks and sulfasalazine), neuropathy, obstructive sleep apnea and prior right hip arthroplasty completed 9 years previously in the setting of avascular necrosis, presented to clinic with a 3-month history of malaise and low-grade temperature of 99°F. He had returned from a mission trip to Honduras 3 months previously. He initially developed a low-grade temperature 1 month after returning, which was followed by unintentional weight loss and night sweats. He had mild congestion and took a course of amoxicillin without improvement in temperature or night sweats. Subsequently, he developed progressive right hip pain. A bone scan was unremarkable. A fever of 103°F prompted hospital admission for evaluation.

The patient reported no additional symptoms at the time of admission and his abatacept was held. He reported drinking only bottled water in Honduras with no adventurous eating. He was born in California but had lived in the Midwest United States much of his life. He is married, monogamous, and had no history of tobacco or recreational drug use. He had traveled to Mexico previously but denied other recent travel. He reported no tuberculosis or animal exposures.

**Figure 1:**
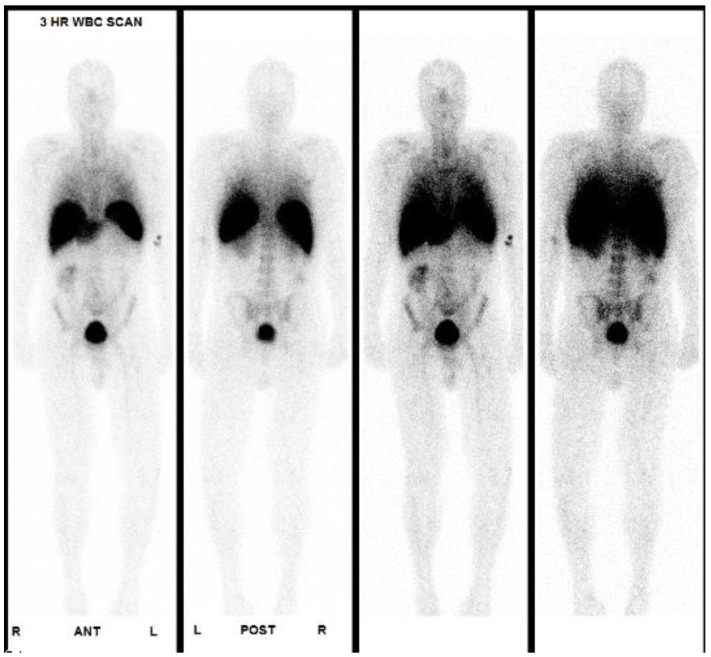
Tagged WBC scan

**Figure 2:**
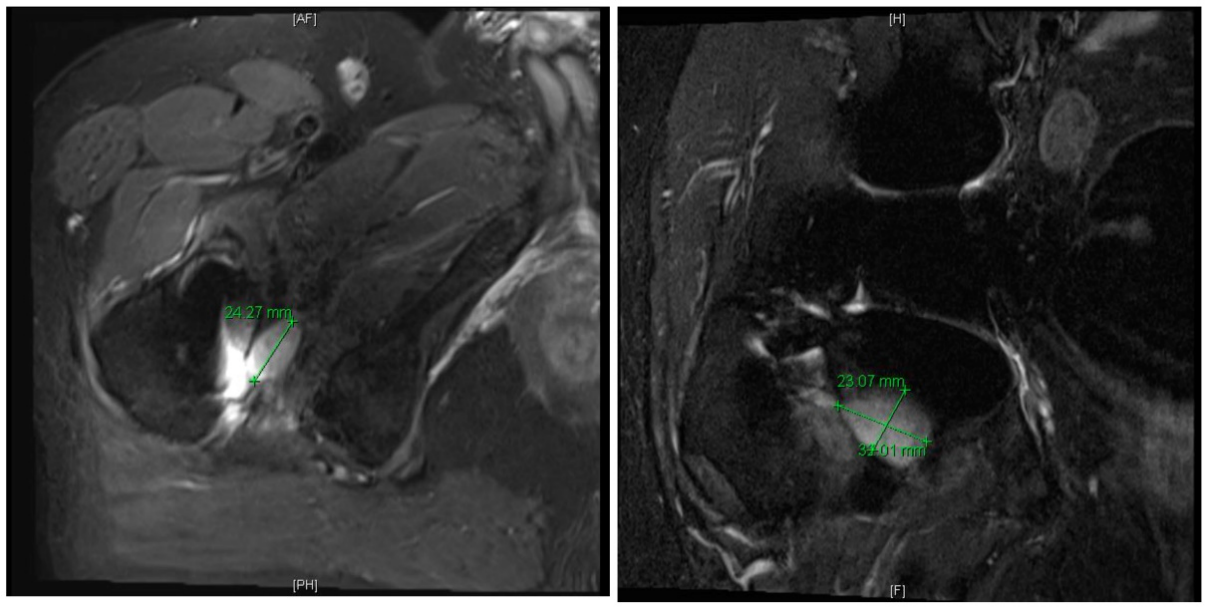
MRI right hip with findings of pseudotumor.

On examination, extremities were atraumatic without synovitis. He had mild discomfort with internal rotation of the right hip. The examination was otherwise unremarkable. Laboratory evaluation revealed a white blood cell count (WBC) of 6.3 K/UL (normal 4.5 - 11.0 K/UL) and hemoglobin of 12.4 grams (normal 13.5 - 16.5 gm/dL). Erythrocyte sedimentation rate (ESR) was 25 mm/h (normal < 20 mm/hr). Liver transaminases were normal. The following infection diagnostics were negative: malaria smear and Ag, HIV ag/Ab, Syphilis Ab, Histoplasma serum Ag and Ab, Coccidiodes serology, Cryptococcal serum Ag, Bartonella and Brucella IgG/IgM, T spot TB, Chikungunya IgG and 2 sets of blood cultures. A chest radiograph and echocardiogram were unremarkable. A 2-view hip radiograph demonstrated right hip arthroplasty without periprosthetic lucency or fracture. The patient underwent a tagged white blood cell scan, which did not demonstrate any increased uptake (Figure 1). An MRI scan of the right hip joint without contrast demonstrated fluid collections medial to the lesser trochanter, consistent with hematoma or pseudotumor (Figure 2). Joint aspirate was grossly purulent. The patient was taken to the operating room for prosthetic explant with antibiotic spacer placement. Antibiotic spacer was ceramic total hip system, which was cemented in with antibiotic-impregnated cement with 5g of Vancomycin and 1.2g of Gentamicin.

Testing from the operated room revealed that the joint aspirate cell count was unreadable as the cells were degenerated. Gram stain of the aspirate was negative for microorganisms. Aspirate routine culture was negative, with no growth at 5 days on aerobic and anaerobic culture. Aspirate grew acid-fast bacteria at 10 days, identified later as MAC. Fungal cultures remained negative. Tissue cultures including femoral head component had negative routine culture and fungal culture. AFB culture of the tissue grew MAC. Blood cultures were negative for mycobacteria at 6 weeks. Macrolide sensitivities were performed and the isolate was found to be clarithromycin susceptible.

Initial broad-spectrum antibiotics were adjusted and he was discharged on intravenous amikacin three times weekly with oral rifampin, ethambutol, and azithromycin. Amikacin was discontinued after 4 weeks of treatment. The remainder of antimicrobial therapy was continued for 12 months. The patient's rheumatoid arthritis treatment included sulfasalazine and hydroxychloroquine, resumed after 3 months of antimicrobial treatment. He started methotrexate treatment 6 months after starting antimicrobial therapy for better joint pain control. Eleven months after treatment initiation, repeat joint aspiration culture was negative. He underwent repeat right THA and tissue cultures were negative. One year following the revision THA he remains free of infection. To date, he remains off biologic immunosuppression.

## Discussion

*Mycobacterium avium* complex is the most common NTM infection in the United States, with many asymptomatic carriers 4 and prevalence higher that than tuberculosis. *Mycobacterium avium* complex is a slow-growing NTM species, most commonly pathogenic in the HIV/AIDS population as disseminated disease or lymphadenitis. It can also colonize the lungs of those with chronic lung diseases such as cystic fibrosis and bronchiectasis, later causing infection characterized by patchy interstitial infiltrates and cavitary lesions. Non-tuberculous prosthetic joint infections are uncommon. Slow-growing NTM infections such as MAC are rare. At the time of writing, only a few case reports of MAC PJI were found in the literature. Most cases occurred in an immunosuppressed patient due to transplant, both renal and cardiac 2,5,6,9. One episode was reported in a patient similar to this patient, who was immunosuppressed due to chronic steroid use for rheumatoid arthritis 6. The patient in the aforementioned case report was also on abatacept for six months prior to presentation. While this patient was initiated on similar antimicrobial regimen (rifampin, ethambutol, and clarithromycin) no surgical explantation was feasible and the patient expired six months after presentation.

With the expanded use of biologics and other immune-modulating therapy in inflammatory diseases and malignancy, the incidence of NTM prosthetic joint infection may increase. Identifying an NTM PJI requires a high index of suspicion for this possibility, as specific mycobacterial cultures are required from tissue/bone in a patient deemed at risk for NTM infection (including use of prednisone, TNF antagonists, tofacitinib and other immunosuppressive therapies) 8. Though MAC infection has not been reported specifically with abatacept (ORENCIA®) screening for latent *Mycobacterium tuberculosis* is recommended prior to treatment initiation given the risk for infection. The pathogenesis of infection for the patient in this case appears to be a late manifestation of possible surgical site infection that occurred secondary to the immunosuppression. Though dissemination via hematogenous spread is a possible mechanism for infection in this case, the patient had no underlying structural pulmonary disease that may have provided a nidus for MAC infection.

There is a lack of consensus on best practices or optimal duration of therapy for NTM PJI, as much must be extrapolated from treatment of pulmonary disease. Susceptibilities for NTM should be obtained. Recommended treatment per guidelines based on limited evidence is use of triple antibiotic therapy (azithromycin/clarithromycin, ethambutol, rifampin) for 6-12 months 4 though guidelines note it is not clear if either three drugs or this time interval is adequate. Evaluation of the response to treatment in localized prosthetic joint infection is invasive, making determinations on appropriate length of treatment arduous. Our patient was monitored routinely with clinic visits and laboratory testing for toxicity to the antibiotic regimen, and re-cultured at the time of definitive surgery, approximately 11 months after starting treatment. His treatment duration was 12 months in total.

## Conclusion

For NTM prosthetic joint infections, recommendations regarding treatment regimens are limited and based upon experiences and outcomes in treating pulmonary and soft tissue infections. As the population ages and more patients undergo treatment with biologics and other immunosuppressing therapies, presumably more patients will be at risk for these types of NTM infections. Increasing numbers of joint replacement procedures will increase potential for PJI. Further investigations into optimal antibacterial therapy choice, intervals, and durations of treatment for NTM, particularly MAC prosthetic joint infections are imperative.
